# Long-Term Effects of Acute Myeloid Leukemia Treatment on the Oral System in a Pediatric Patient

**DOI:** 10.2174/1874210601812010230

**Published:** 2018-03-30

**Authors:** Saturnino Marco Lupi, Arianna Rodriguez y Baena, Gabriele Cervino, Claudia Todaro, Silvana Rizzo

**Affiliations:** 1 Department of Clinical, Surgical, Pediatric and Diagnostic Sciences, School of Dentistry, University of Pavia, P.le Golgi 2, 27100 Pavia , Italy; 2 Department of Biomedical and Dental Sciences, Morphological and Functional Images, School of Dentistry, University of Messina, Policlinico G. Martino, Via Consolare Valeria, 98100 Messina, Italy

**Keywords:** Acute myeloid leukemia, Dental anomalies, Children teeth, Bone marrow transplantation, Long-term effects, Dental implants

## Abstract

**Introduction:**

Acute Myeloid Leukemia (AML) in pediatric patients is a serious disease, although, for the subgroup of patients who receive proper treatment, a long-term survival rate above 50% is typical. The cycles of chemo- and radiotherapy used to treat AML can impair dental development.

**Case Report:**

Herein, we describe the oral condition of a 25-year-old male patient treated for AML with chemo- and radiotherapy from 5 to 7 years of age; his AML has remained in remission for the past 18 years. He had lost only one permanent tooth, but the remaining teeth demonstrated serious deformations and radicular hypoplasia. Two teeth required immediate extraction and subsequent replacement by implant-supported crowns. We found that the decayed, missing, filled teeth (DMFT) index was not representative of the real oral condition. Here, we report the full case and provide a brief review of the literature.

**Conclusion:**

Antitumor treatment of pediatric leukemia can induce total impairment of dental development and function. These adverse effects may become clinically evident many years after the resolution of cancer, and can be significantly detrimental to the patient’s quality of life.

## 1 INTRODUCTION

Acute leukemic diseases are clonal pathologies characterized by an arrest of the maturation and an increase in the proliferation of hematological progenitor cells that in normal situations would differentiate into mature cells [1].

The symptoms of leukemia depend on the proliferation of neoplastic cells and on their influence on non-neoplastic cells. Erythroblast reduction leads to strain, anemia, pallor and tiredness; granulocyte reduction allows persistent infection and fever and platelet reduction produces petechiae, ecchymosis and spontaneous bleeding. Leukemic cells can invade the spleen, lymph nodes, central nervous system, and the gingiva, and sometimes induce bone pain and arthralgia [2].

The leukemias are the most frequent form of pediatric cancer, representing 27% of the malignant pathologies in the USA. Acute leukemia takes on two different forms: Acute Lymphoblastic Leukemia (ALL), which represents 21% of malignant tumors, and Acute Myeloid Leukemia (AML), which represents 5% [3]. The incidence rates in US pediatric patients are 41.9 cases/year/million population for all leukemias, 31.9 cases/year/million population for ALL and 7.5 cases/year/million population for AML [3]. Notably, a male-dominant sex difference in ALL has not been found in AML [3]. The two forms also diverge regarding the age of onset, such that AML is more likely to occur from birth to age 4 or from ages 15-19. Moreover, pediatric leukemias have demonstrated a minor and not statistically relevant increase in incidence in the last few years [3].

Acute leukemia is treated *via* chemo- and radiotherapy [4]. The prognosis for AML in pediatric patients has improved greatly in the last 4 decades, with a remission rate of over 80% and survival rates increasing from 3% during the ’70s to over 60% currently [5-8]. Those improvements are linked to a better comprehension of the molecular and cellular biology of the disease, more aggressive chemotherapy, stabilization of remission and improvement of support treatments such as blood transfusions and immune system support [6, 9-11].

Oral signs of leukemia appear precociously in two-thirds of all patients. Although not specific, signs that should alert physicians include the simultaneous presence of different lesions, the absence of pathogenic noxae, and the timing of and size at onset [12]. The most frequent oral signs of leukemia are gingival hypertrophy, temporomandibular joint arthritis, osteolytic lesions in the mandible, hematomas, ulcers and opportunistic infections [13-15].

The effects of chemo and radiotherapy on the orofacial area depend on the age of the patient at the beginning of treatment and the chronic irradiation dose [4]. Dental alterations are identified in approximately 80 and 90% of all patients at the diagnosis of children of age 1-6 and 7-12 years-old, respectively [9]. Anti-neoplastic therapies can lead to enamel malformation (discoloration and hypoplasia), as well as to radicular anomalies such as resorbed or tapered roots, delay of root formation, early apical closure, delayed dental development or dental impaction, dental shape anomalies (microdontia, macrodontia, taurodontia) and anomalies in numbers (hypodontia, supernumerary teeth) [4, 16, 17]. The anomalies depend on the odontogenetic phase during which the therapy was conducted. Therefore, an accurate knowledge of dental development by clinicians is fundamental to understanding the timing and nature of anti-neoplastic treatment-related dental anomalies and their correlation with the onset age.

## 2 CASE REPORT

In February 2016, a male patient, 25 years of age, came to the Dental Service of the Department of Clinical Surgical, Diagnostic and Pediatric Sciences of University of Pavia, reporting dental mobility and pain in the superior second premolar on the right side.

After a complete anamnesis, the first clinical examination showed moderate-to-severe dental mobility in all teeth except for the maxillary and mandibular first molars.

**Fig. (1):**
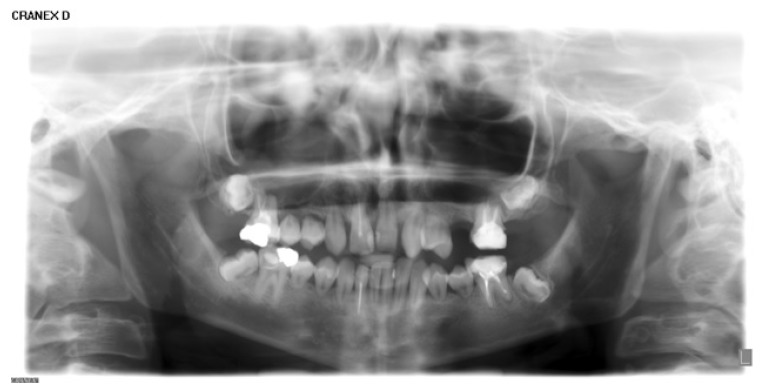
Initial examination of the patient *via* panoramic radiography.

At this point, a panoramic radiograph was acquired (Fig. **1**).

The patient’s history is resumed in Table **1**.

In January 2009, at the end of the therapeutic process, the oncohematologist who successfully treated the patient reported the following side effects caused by the anti-neoplastic treatment regimens:

- Hypometria of the lower left limb.
- Short stature caused by reduced growth hormone secretion (treated with hormone replacement therapy).
- Reduced lacrimal secretion;
- Minor restrictive dysventilation syndrome secondary to the GVHD.
- Hyperplastic thyroid nodules caused by the treatment received.

Because of the risk from the thyroid nodules, the patient underwent a total thyroidectomy in May 2009, and is currently on levothyroxine for the subclinical hypothyroidism and Testoviron® for the treatment of hypogonadotropic hypogonadism.

Clinically, the dental crowns presented with normal dimensions and calcification. Upon our examination, though, all his present teeth showed anomalies in radicular shape, such that the roots were decapitated and seriously hypoplastic. We did not know if the current radicular conformation was present at the end of his anti-neoplastic therapy, or if it changed into the current conformation afterward. The second superior premolar on the left side self-exfoliated without clinical intervention.

The patient presented with a Decayed Missing Filled Teeth (DMFT) index of 39.29%. When including the number of dental elements affected by aberrations, and not only DMF scores, the percentage increased to 100%. The two lateral incisors were missing because of agenesis, as opposed to extraction.

The two premolars on the superior right side were extracted; however, it is likely that in a short time, the patient will require further dental treatment related to dental damage by the previous anti-neoplastic therapy.

**Fig. (2):**
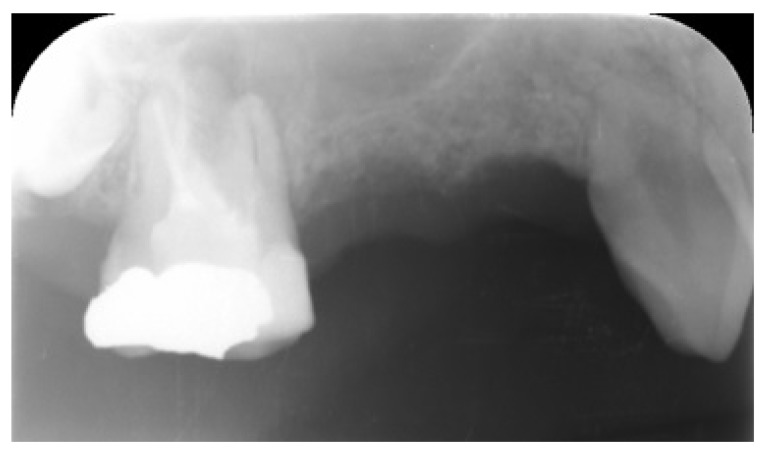
Radiograph after tooth extraction and healing before dental implants.

**Fig. (3):**
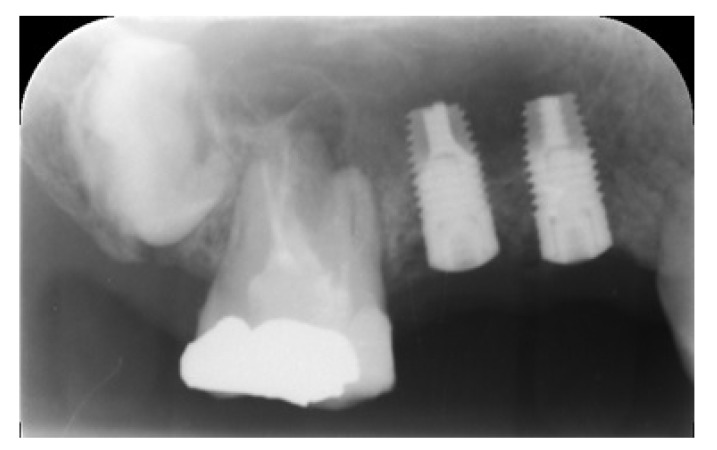
Post-surgery radiograph demonstrating the screw-type implants.

Three months after dental extraction (Fig. **2**), two screw-type implants (4 mm width and 10 mm length, Nobil Bio Ricerche S.r.l, Portacomaro (AT), Italy) with an etched and sandblasted surface were placed *via* a conventional submerged surgical technique [18], with a crestal sinus lifting and no bone graft (Fig. **3**). Stitches were removed after 14 days and during healing, no adverse event occurred.

**Fig. (4):**
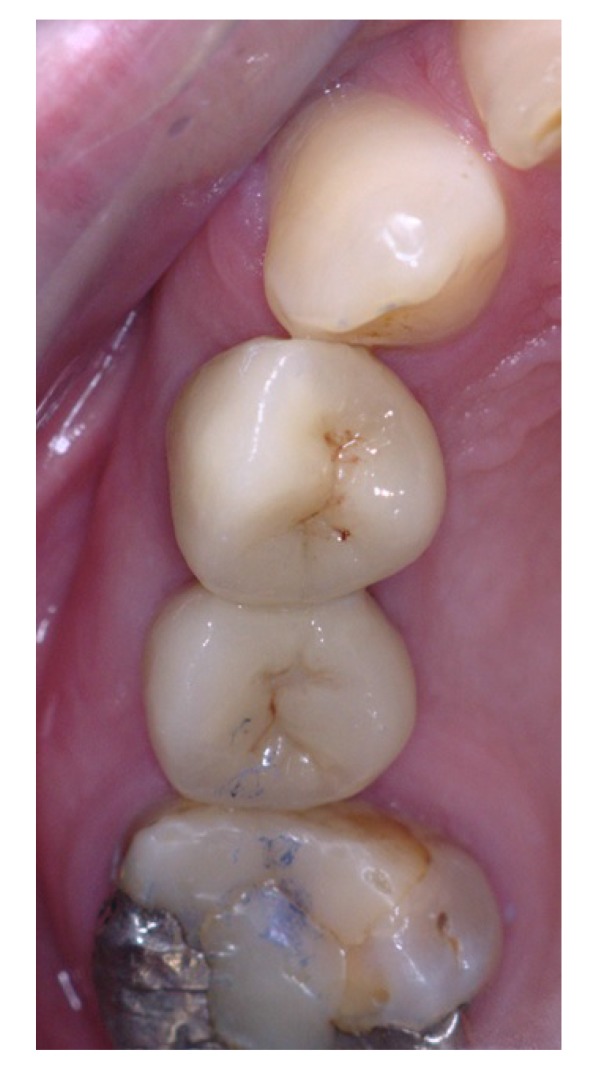
Prosthetic rehabilitation with fixed crowns. Occlusal view.

**Fig. (5):**
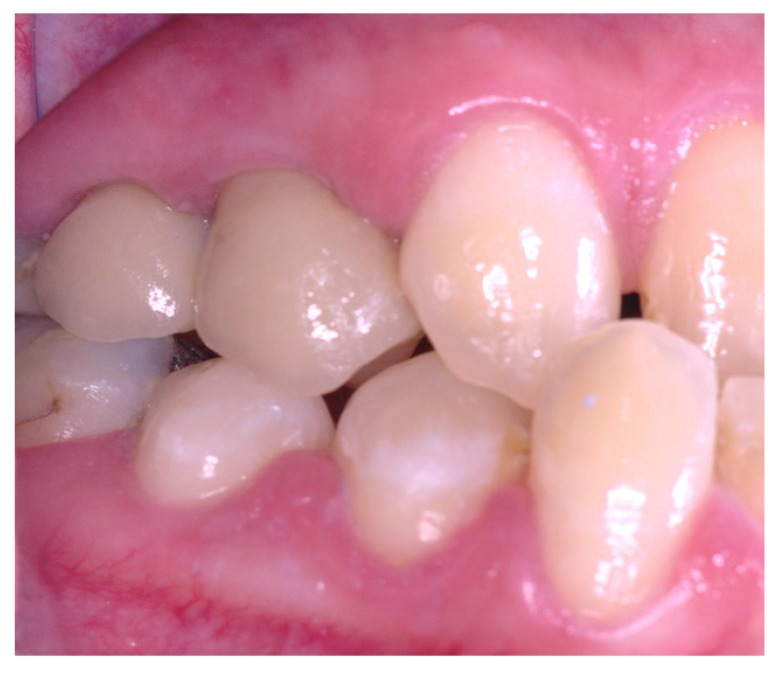
Prosthetic rehabilitation with fixed crowns. Lateral view.

**Fig. (6):**
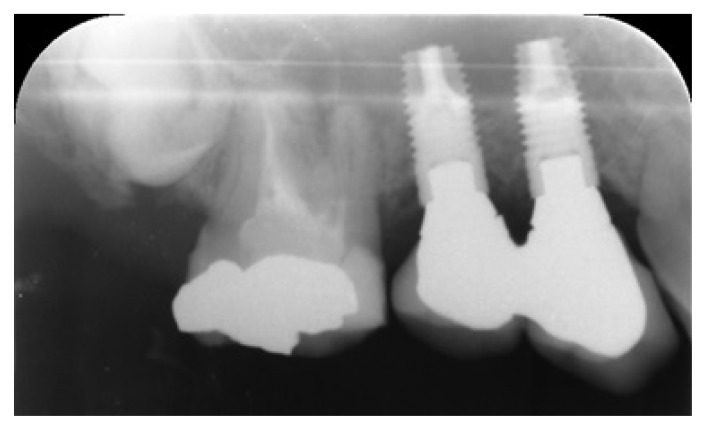
Ray six months after prosthetic load. The bone surrounding the implants appears stable.

Three months after placement of the implants, a second surgery was conducted and routine prosthetic procedures were performed to finally fix the crowns for rehabilitation (Figs. **4** , **5**). The patient followed a strict protocol of oral hygiene [19]. Six months after prosthetic rehabilitation, a follow-up radiograph showed a stable bone surrounding the implants (Fig. **6**). No sign of peri-implant soft tissue inflammation was present. Written informed consent to use the patient’s data was obtained. Patient’s data were reported anonymously.

## 3 DISCUSSION

At present, the survival rate for AML with treatment is over 50% [19]; the patient reported herein is still considered free of pathology, 10 years after the last anti-neoplastic treatment. Furthermore, he has not shown any signs of recidivism in the last 18 years. Since more patients with AML are expected to live in adulthood, this case will assist in the future evaluation of AML survivors in terms of the long-term dental effects of anti-neoplastic treatment. It is also possible that the information provided by this case will be applicable to other juvenile cancer treatments, though future work is needed to assess this accurately. Moreover, it is not common to diagnose dental lesion related to pediatric treatment for leukemia, but it is possible that oral diagnosis is underestimated due to lack of knowledge.

AML is infrequent in pediatric patients, primarily presenting in people older than 50, with a spike around the age of 70 [8]. In children, its onset is most frequent before the age of 4 and after the age of 15 years [20].

In this case, the monocytic AML diagnosis was made initially at the age of 5 years, and the patient was treated with the modified AIEOP LAM 92 protocol (idarubicin, cytarabine, and etoposide during the induction cycles, and cytarabine during consolidation). He also underwent a conditioning regimen of busulphan, cyclophosphamide, and melphalan, three alkylating agents potentially capable of killing dormant preleukemic stem cells [3]. The original AIEOP protocol includes cranial irradiation and auto/allotransplantation of bone marrow [21]. In this case, the patient was not initially treated with radiotherapy; 2 years after the first diagnosis, and following the second recidivism, the patient was treated with total-body radiotherapy. Pediatric patients treated for AML frequently show recidivism [22], and therefore require multiple cycles of anti-neoplastic therapy. In this case, AML was diagnosed at the age of five years, with treatments for recidivism at six and seven years using chemo- and radiotherapy as well as bone marrow transplantation. In this age range, the permanent teeth normally undergo the development phase; during this time, the patient underwent three anti-neoplastic therapeutic cycles.

All of the patient’s teeth presented with radicular shape anomalies, demonstrating decapitation and severe hypoplasticity. As mentioned above, we do not know if the radicular conformation observed at our examination remained unchanged since the time his teeth developed, or if it changed following a radicular reabsorption occurring after the complete formation of roots. The inferior incisors and first molars were the teeth that presented with the least radicular shape alterations; the roots had developed for over half of their regular length. Notably, those are the first permanent teeth to erupt, so it is likely that their roots were almost completely formed at the moment of the pathogenic insult. The canines and central superior incisors showed roots shorter than half their usual length. All of his premolars presented with only a hint of the roots, and the second molars demonstrated no radicular portion, and they were retained. The wisdom teeth were not visible. The severity of the dental-facial anomalies caused by anti-neoplastic treatment is linked to the age of the patient at the start of the treatment and to the administration of cranial radiotherapy [8].

The literature reports that children treated before the age of 5, presented with the most serious dental anomalies, because underdeveloped teeth have a higher risk of developing deformities compared to the already developed ones. Chemotherapy without cranial irradiation causes less dental aberrations. The effects of radiotherapy on dental development are well documented: doses of 2000-4000 cGy induce dental or radicular hypoplasia, radicular reabsorption, microdontia, or abnormal radicular morphology. Radiotherapy administered before morphodifferentiation and calcification can prematurely halt tooth germ development. In the later development phases, dental aberrations and developmental terminations have been reported [4].

Even though in this case the patient was treated for AML after the age of 5, the long-term side effects of the chemo and/or radiotherapy caused anomalies in all his permanent teeth that, although they are still mostly present in the oral cavity, showed such compromising anomalies as to demand extraction and the precocious loss of teeth. Due to the development stage of incisors, probably the inhibition of dental growth occurred around the age of six years, *i.e*. before radiotherapy. In this situation, the DMFT index does not seem to be informative, since it is related to clinical and not radiographic aspects; this leads to an underestimation of the severity of the dental situation. We feel that the long-term side effects of anti-neoplastic treatment on dental health should be taken into consideration by the Italian legislature for the evaluation of handicap status.

Dental long-term effects of anti-neoplastic therapy in childhood are poorly reported [4]. Frequently, reports on long-term complications and outcomes of such therapies simply do not consider dental development [23]. Previously [24, 25], dental lesions similar to those presented herein have been reported, although the report described a female patient treated for Hodgkin's lymphoma in the neck region. Moreover, root abnormalities were localized to the lower jaw, indicating that the pathogenic insult was the radiotherapy and not the chemotherapy, which acts systemically. Further supporting this hypothesis, the root abnormalities were localized to at least the incisors and molars.

Implant therapy is a good treatment option [26] in healthy individuals, but in adult patients who have had pediatric leukemia, it is poorly reported [18]. In a case of a healthy individual like this, a major sinus lift or bone regeneration technique could be employed [27-31]. Those approaches were excluded here due to a lack of reporting on those specific situations. The primary objective in choosing the actual therapeutic option was to provide rehabilitation with the minimum risk of failure, considering the medical history of the patient. Moreover, implant loading can influence osseointegration at the bone regeneration site [32]; this case showed a stable bone surrounding the dental implants 6 months after the rehabilitation.

## CONCLUSION

We treated a 25-years old male patient treated with chemo- and radiotherapy for AML 18 years prior to his dental examination in our clinic, during the development of his permanent teeth. The repeated anti-neoplastic therapies cured the AML, but induced long-term adverse effects that caused complete impairment of the development of his permanent teeth, and the premature loss of several of them.

Those issues, because of their gravity and the early manifestation, greatly compromised the patient’s quality of life.

## ETHICS APPROVAL AND CONSENT TO PARTICIPATE

Not applicable.

## HUMAN AND ANIMAL RIGHTS

Not applicable.

## CONSENT FOR PUBLICATION

Written informed consent to use the patient’s data was obtained. Patient’s data were reported anonymously.

## Figures and Tables

**Table 1:** Patient's history.

November 1996	Diagnosis of monocytic AML (age 5), by the Department of Pediatric Oncohematology of Policlinico San Matteo of Pavia;
-	Chemotherapy for the diagnosed AML, according to the modified AIEOP LAM 92 protocol;
March 1997	Diagnosis of medullary recidivism (age 6) refractory to the above chemotherapy protocol upon its re-introduction;
April 1997	Allogenic bone marrow transplantation from an HLA-adequate donor (the patient’s brother), after conditioning via busulfan, cyclophosphamide, and melphalan administration;
April 1998	Diagnosis of new recidivism at the rear-peritoneal abdominal level (age 7);
July 1999	Allogenic bone marrow transplantation from patient’s brother, after a myeloablative treatment via total-body irradiation combined with cyclophosphamide and thiotepa;
January 2009	Diagnosis of AML-free (age 18) after reaching the 10-year disease-free checkpoint.
